# A Decentralized Fuzzy Rule-Based Approach for Computing Topological Relations between Spatial Dynamic Continuous Phenomena with Vague Boundaries Using Sensor Data

**DOI:** 10.3390/s21206840

**Published:** 2021-10-14

**Authors:** Roger Cesarié Ntankouo Njila, Mir Abolfazl Mostafavi, Jean Brodeur

**Affiliations:** 1 Centre for Research in Geospatial Data and Intelligence, Department of Geomatics Sciences, Université Laval, Quebec, QC G1V 0A6, Canada; 2 GéoSémantic Research, Sherbrooke, QC J1L 1W8, Canada; recherchegeosemantic@videotron.ca

**Keywords:** sensor networks, decentralized spatial reasoning, fuzzy spatial relations, dynamic phenomena, fuzzy-crisp objects

## Abstract

Sensor networks (SN) are increasingly used for the observation and monitoring of spatiotemporal phenomena and their dynamics such as pollution, noise and forest fires. In multisensory systems, a sensor node may be equipped with different sensing units to observe and detect several spatiotemporal phenomena at the same time. Simultaneous detection of different phenomena can be used to infer their spatial interactions over space and time. For this purpose, decentralized spatial computing approaches have shown their potential for effective reasoning on spatial phenomena within a sensor network. However, in most cases, spatial extents of continuous dynamic phenomena are uncertain, and their relations and interactions cannot be inferred by the existing approaches at the sensor node level. To address this limitation, in this paper, we propose and develop a decentralized fuzzy rule-based spatial reasoning approach to depict the spatial relations that hold between two evolving spatial phenomena with fuzzy boundaries. The proposed method benefits from a more adapted fuzzy-crisp representation of dynamic phenomena observed by SN where each vague phenomenon is composed of five distinguished zones including the kernel, conjecture and exterior zone and their boundaries. For each detected phenomenon, a sensor node will report one of these zones based on its location. Aggregation of the information reported from the sensor nodes allows reasoning on spatial relations between the observed phenomena and their evolution. Such spatial information provides users with more valuable near real-time information on the state of different phenomena that can be used for informed decision-making.

## 1. Introduction

In recent decades, sensors have been continuously improved and have become smaller, cheaper and more intelligent [1]. These technologies are increasingly used for many applications, such as in military, healthcare, area surveillance, environmental monitoring, security, etc. [2]. Sensors help in remotely measuring the physical properties of phenomena of interest in order to detect their presence at a given location and time [3]; they allow users to derive and maintain an accurate and up-to-date digital picture of continuously changing real-world phenomena which may be either discrete (e.g., people movement) or continuous (e.g., temperature, humidity). In particular, spatiotemporal continuous phenomena monitored by sensor networks are inherently fuzzy and uncertain [4], and hence their spatial shape is ultimately vague. Fuzzy-crisp spatial objects promoted in [5] represent and model vague-shape phenomena. In this model, the geometry of an object is composed of a kernel and a conjecture part. The kernel part definitely belongs to the vague object but one cannot say with certainty whether the conjecture part, which is considered as the broad boundary, belongs to the vague object. Understanding the complexities of the detection, monitoring and representation of such vague spatial phenomena and their dynamics and their interactions with other similar objects using sensor networks is a very challenging task.

Among these complexities, the detection and monitoring of the evolving relations and interactions between vague-shaped dynamic phenomena is very important for informed decision-making. Hence, topological relations are the predominant backbone of qualitative spatial reasoning focusing mostly on objects that are embedded in R2 [6]. Topology is the study of the relative positioning of two geometric objects [7]. The division of the internal structure and external part of geographical entities is the premise of the analysis of topological relations. Currently, most of the existing geographic information systems for representing spatial relations are developed for crisp objects [8], which are less adapted for the representation and analysis of real-world situations about many environmental phenomena with fuzzy boundaries. In contrast with such tools, sensor observations break the internal and external divisions of the spatial extent of a vague phenomenon into more fine spatial units, adding therefore a second level of vagueness in sensor observations. Analyzing spatial relationships that hold between monitored phenomena from sensor measurements therefore becomes more challenging. For instance, using sensor data observed for two vague-shaped phenomena A and B, we need to express relations such as the phenomenon A is nearly overlapping phenomenon B if the kernel part of A overlaps the conjecture part of B or the conjecture part of A overlaps the conjecture part of B or the kernel part of B overlaps the conjecture part of A; such information would be important for spatial decision support systems (SDSS).

Formal representation of spatial relations between objects is an important part of theories of spatial data [9]. Many computing models have been developed for spatial modeling and reasoning about geospatial phenomena and spatial relations. The most important examples of these models include the 4-intersection model (4IM) [10], 9-intersection model (9IM) [11], Region Connection Calculus (RCC) [12], or calculus-based model (CBM) [13]. RCC sets of relations such as RCC5 and RCC8 were originally developed for ideal regions, not subject to imperfections such as vagueness or fuzziness which are found in many applications requiring geographic analysis [14]. The RCC model has been popularly adopted by qualitative spatial reasoning, but it lacks formal description and the relations are hard to calculate [15]; RCC is therefore difficult to extend and use to represent topological relations among fuzzy regions. On the other hand, considering the vague nature of fuzzy regions and related complexities involving their spatial relations, Li and Li [14] found RCC5 or RCC8 corresponding composition tables too arduous and error-prone. Such reasoning becomes even more complex considering the necessity of on -the-fly reasoning on multi-sensor data obtained from continuously evolving vague-shaped phenomena. 

When equipped with different sensing devices, sensor networks allow multiple calculations and combination of stimuli about more than one phenomenon, as presented in SSN ontology [16]. Simultaneous detection of two phenomena in a sensor node (A and B) reveal the intersection of A and B in that node, while their local disjunction may be revealed by the single detection of A or B. In such a partitioned topological space, the Intersection Model (IM) as an integrated model [17] seems appropriate for the description of the relations between A and B. However, because sensor observation offers a high level of granularity in describing a scene, particularly about spatial relations among fuzzy-crisp regions with broad boundaries, the 4IM and 9IM presented in [10,11] cannot describe the spatial relations that hold between such regions. 

In contrast to 4IM and 9IM models, the $I_{5\times 5}M,$ which is an extension of the 9IM model, presents an interesting alternative solution for the representation and analysis of the spatial relations between vague-shaped spatial objects. This model is presented by a 5 × 5 matrix in which each of the 25 distinct elements can represent, on a Boolean base, a specific intersection between two fuzzy-crisp objects, each composed of five distinct parts [18]. Then, the 5 × 5 Intersection Model $\left(I_{5\times 5}M\right)$ can be built based on the intersection between the parts of two vague-shaped phenomena inferred over the sensor network to describe the spatial relation that holds between them. 

Hence, in this paper, we propose and develop a novel decentralized fuzzy rule-based spatial reasoning approach based on the ($I_{5\times 5}M$) to depict the spatial relations that hold between two phenomena with fuzzy boundaries monitored by a sensor network. In this approach, sensor data are used to describe five distinct states for each sensor with respect to each spatially evolving continuous phenomenon with fuzzy boundaries. The structure and values in $I_{5\times 5}M$ are used to describe and depict spatial relations that hold between monitored phenomena at a given time with the aim of better supporting the spatial decision-making process. In this approach, partial and local spatial relations between monitored phenomena are deducted from sensor observations of individual sensor nodes and their collaboration with linked neighboring nodes, while the spatial relations as a whole and their changes are inferred overall to the SN extent. The proposed approach is then applied to a case study and its advantages and limitations are discussed. 

The remainder of this paper is organized as follows. Section 2 revises some key concepts on spatial reasoning and computing models for topological relations, with special attention paid to the models describing spatial relations between objects with vague-shaped and ill-defined boundaries. Section 3 presents the framework of the proposed approach in computing qualitative spatial information depicting fuzzy relations that hold between phenomena with fuzzy boundaries monitored by sensor networks. This approach is then formally described for its implementation, the results of which are presented in Section 4 while testing its applicability over the 44 scenes of fuzzy spatial relations. Section 5 draws conclusions and identifies future research works.

## 2. Background on Spatial Reasoning and Computing Models for Topological Relations

Spatial relations between objects are defined based on their relative positions. These relations are widely used by the query languages for spatial data retrieval and analysis in geographic information systems (GIS) [19]. Spatial relations are commonly grouped into topological relations, direction relations and metric relations [20,21]. Topological relations are the predominant backbone of qualitative spatial reasoning, focusing mostly on objects that are embedded in R2 [6] and more recently on three-dimensional space. Topology is the study of the relative position of two geometric objects [7] at a given time. The division of the internal and external spaces of geographical entities is the premise of the analysis of topological relations; currently, most division methods are crisp [8], which does not conform to the real-world situations where many environmental phenomena have fuzzy boundaries (e.g., air pollution, heat islands, magnetic fields, storm intensity) [22].

Spatial modeling and reasoning about real-world phenomena from sensor data has received a lot of attention in recent years [23]. Many computing models have been developed for spatial modeling and reasoning about geospatial phenomena and spatial relations, among which the calculus-based model (CBM) [13,18], the Intersection Model (IM) is the most popular formal and extensible model with different versions. However, many challenges regarding modeling and analysis of the large-scale phenomena with ill-defined boundaries from sensor data still persist. The fuzzy-crisp region model proposed by Paul and Schneider [5] and adopted in [24] is an example of model for more realistic representation of fuzzy phenomena. This model, which is, to some extent, similar to the α-cuts model [25], is composed of kernel and conjecture parts. The kernel part definitely belongs to the vague object. However, one cannot say whether the conjecture part, which is considered as the broad boundary of the vague object, belongs to it with certainty.

### 2.1. The Region Connection Calculus (RCC)

Because topological relations constitute an important facet of how humans perceive spatial configurations, a large proportion of the spatial information conveyed in natural language discourse is related to topology. For instance, one may like to find out, if two regions are adjacent, if one is contained within the other or if they overlap. The Region Connection Calculus (RCC) method has been proposed as a means to model such topological relations [12]. Reference [26] suggests that it is possible to effectively compute spatial relations with a quite expressive set of spatial relations using the RCC model. This allows building a wide range of decision procedures based on inferred topological relations. RCC is a qualitative spatial representation based on a simple primitive relation of connection between regions. Relationships are defined by means of connection relation $C\left(A,\;B\right)$ which holds between phenomenon A and B when A and B share a common point. In sensor networks, if A and B are jointly sensed by the same sensor node, this means A and B are present at this particular point and may have several possible relations. 

**Figure 1:**
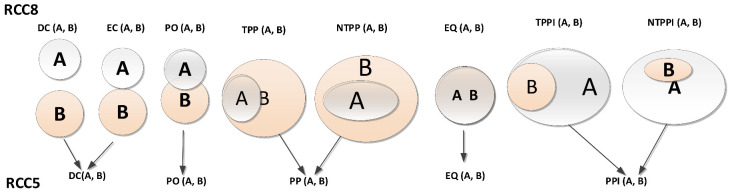
Illustration of RCC5 and RCC8 set of topological relations.

Two versions of RCC exist; the RCC-5 and RCC-8 schemes consist of five and eight basic topological relations, respectively. The two sets of topological relations (RCC-5 and RCC-8) were compared by Baode and Qi [15], as shown in Figure 1. In RCC-5 the influence of boundaries is ignored [15], leading to the five spatial relationships: DC(A, B) for A and B are disconnected; PO(A, B) for A and B partially overlap; PP(A, B) and PPI(A, B) for A is a proper part and inverse proper part of B, EQ(A, B) for A is equal to B, whilst considering the effect of boundaries as is the case in RCC-8, three other relations are considered: EC(A, B) for A is externally connected to B, TPP (A, B) and TPPI(A, B) for A is a tangential proper part or inverse tangential proper part of B. 

RCC sets of relations, such as RCC5 and RCC8, were originally developed for crisp regions, not subject to imperfections such as vagueness or fuzziness which are found in many applications requiring geographic analysis [14]. The RCC model has been popularly adopted by qualitative spatial reasoning, but it lacks formal description and is hard to calculate [15]; it is therefore difficult to extend RCC and apply it to compute topological relations among fuzzy regions in sensor networks characterized by limited distributed computing resources. Binary complements are helpful but still sound reductive [27]. The use of a single connection primitive ($C\left(A,B\right)$) for reasoning about spatial relationships, as in the case in basic RCC, requires further fuzzy reasoning rules to infer spatial relations that hold between monitored phenomena with vague spatial shape [28] information on broad boundaries in reasoning about topological relations between regions, which may be useful for SDSS, as shown in RCC-8 for simple crisp regions. 

### 2.2. The Intersection Model (IM)

Based on point-set topology, the first IM was formerly presented in [10], based on which many other IM versions have been developed over time. The followings are the most prominent IM:

- the 4-intersection model (4IM), which has been extended to cope with various spatial considerations into many IM releases[10],
- the 6-intersection model 6-IM [15],
- the 9-intersection model (9IM) [11],
- fuzzy 9-Intersection Model (F9-IM) [29],
- the 16-intersection model (16-IM) [18,30],
- the 25-intersection model (25-IM) [20,31].

In 4-IM, spatial objects are divided into boundary and interior and describes the topological relations between two spatial objects using a quaternion matrix, represented as follows:

(1) $$R4IM=\left[\begin{array}{cc} A\cap B & A\cap \partial B\\ \partial A\cap B & \partial A\cap \partial B \end{array}\right]$$

where A and ∂A denote the interior and boundary of the object A, and B and ∂B denote the interior and boundary of B, respectively. The intersection between two crisp sets may be either non-empty (1) or empty (0), so a 4-intersection model can describe 16 topological relations, among which only 8 topological relations between two crisp regions are significant [29]. These eight topological relationships are: disjoint, meet, overlap, contain, cover, coveredBy, containedBy and equal. These relations correspond to RCC-8 relations: DC, EC, PO, NTPP, TPP, TPPi, NTPPi, EQ, respectively [32]. The 4IM has a limited ability to distinguish topological relations because it is easy to confuse relations (for example between line/line relations and line/region) [31]. Furthermore, there is no consideration about the eventual broad nature of boundaries.

The 9IM was proposed in [11], where the exterior of objects is considered in addition to their interior and border compared to 4IM. An R9IM for modeling topological relations between simple crisp regions can be represented as follows:

(2) $$R9IM=\left[\begin{array}{cc} \begin{array}{c} \begin{array}{cc} A\cap B & A\cap \partial B\; \end{array}\\ \begin{array}{cc} \partial A\cap B & \partial A\cap \partial B \end{array} \end{array} & \begin{array}{c} A\cap B^{-}\\ \partial A\cap B^{-} \end{array}\\ \begin{array}{cc} A^{-}\cap B & A^{-}\cap \partial B \end{array} & A^{-}\cap B^{-} \end{array}\right]$$

where, A, ∂A and $A^{-}$. represent the interior, boundary and the exterior of the object A, and B, ∂B and $B^{-}$ represent the interior, boundary and the exterior of the object B, respectively. Considering the interior, boundary and the exterior of objects gives place to 512 ($2^{9}$) possible topological relations. However, most of the topological relations determined by the 9-intersection model make no sense if the physical reality of 2D space is considered [29].

**Figure 2:**
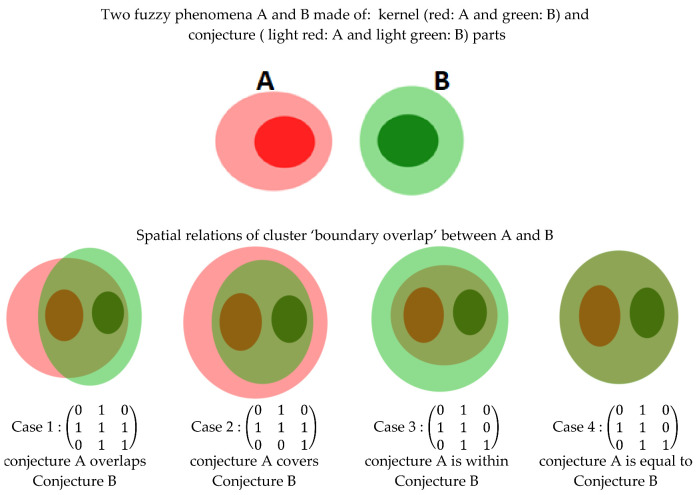
Cluster “boundary overlap” of spatial relations between regions with broad boundaries (conjecture part) modeled using the 9IM with poor specification on topological relations.

Clementini and Di Felice [33] have considered the broad nature of a region boundary and modeled 44 cases of significant spatial relationships that hold between two regions with broad boundaries. In their approach, they build a conceptual-neighborhood graph for regions with a broad boundary. Then, the 44 significant cases of topological relations are clustered in 14 groups of qualitative spatial relations [33] using a topological distance concept. Topology distance is a major argument used in building clusters and analyzing topology consistency in the defined spatial relation [34]. For instance, let us consider the cluster of spatial relations named “boundary overlap” in [33] as shown in Figure 2. In this cluster, we have four relations defined between objects A and B (boundary of A overlaps that of B (case 14); boundary of A contains that of B (case 15); boundary of A is within that of B (case 16); boundary of A is equal to that of B (case 17)). As we can see, the differences in these relations between boundary of A and that of B are ignored in the cluster level even though these differences may be of major importance for SDSS.

A vague-shaped region considered in the 44 topological relationships presented in [33] is based on the egg-yolk model of fuzzy regions [35]. Tang et al. [18] have considered spatial regions with broad boundaries with settings in which the yolk (kernel) part may be out of the white (conjecture) and have shown possible settings of a simple fuzzy region. Based on this consideration, the 9IM seems irrelevant to model spatial relationships between such vague-shaped regions, and Tang et al. [18] suggested the use of a 4 × 4IM (16IM) for modeling 152 cases of realizable spatial relations, but qualitative specifications about these relations require supplementary analysis over the resulting 4 × 4IM. It is in the same order that the Dimensionally Extended 9IM is developed as an appropriate model for analyzing topological relationships between complex regions with broad boundaries [36]. The dimensional extended 9-intersection model (DE-9IM) extended the 9IM by considering the dimensions of inferred intersections; DE-9IM can distinguish more detailed topological relations compared to the 9IM [21]. 

It is important to mention that applying such a model for reasoning on spatial relations of vague-shaped objects from sensor data is more challenging. This is because it is difficult to effectively extract the spatial extent of the vague shaped phenomena from sensor observations due to their limited sensing ranges. This limits the effective computation of the dimension of the intersection among poorly identified parts of observed phenomena.

## 3. Computing and Modeling Fuzzy Spatial Relations Using $I_{5\times 5}M$ in SNs

**Figure 3:**
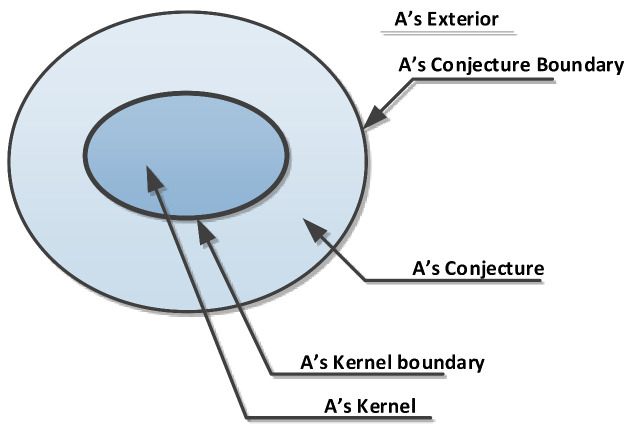
Topological parts of a simple fuzzy-crisp region.

As mentioned earlier, we consider that the fuzzy-crisp region model is more appropriate for realistic reasoning about environmental phenomena with vague or fuzzy boundaries. According to this model, a simple fuzzy-crisp region is composed of its kernel and conjuncture regions and can be expressed using five distinguished topological parts [18], as presented in Figure 3. 

Let us consider two fuzzy-crisp regions A and B. Let us also consider $A^{1},\partial A^{1},\;A^{0},\;\partial A^{0}\;\mathrm{and}\;A^{-}$ to be the kernel, boundary of the kernel, conjecture, boundary of the conjecture and exterior of A, respectively, and $B^{1},\partial B^{1},\;B^{0},\;\partial B^{0}\;\mathrm{and}\;B^{-}$ to be the kernel, boundary of the kernel, conjecture, boundary of the conjecture and exterior of B, respectively. The relations between A and B can be built using the 25-Intersection model $\left(I_{5\times 5}M\right)$ model as follows:

(3) $$I_{5\times 5}\left(A,B\right)=\left[\begin{array}{ccccc} A^{1}\cap B^{1} & A^{1}\cap \partial B^{1} & A^{1}\cap B^{0} & A^{1}\cap \partial B^{0} & A^{1}\cap B^{-}\\ \partial A^{1}\cap B^{1} & \partial A^{1}\cap \partial B^{1} & \partial A^{1}\cap B^{0} & \partial A^{1}\cap \partial B^{0} & \partial A^{1}\cap B^{-}\\ A^{0}\cap B^{1} & A^{0}\cap \partial B^{1} & A^{0}\cap B^{0} & A^{0}\cap \partial B^{0} & A^{0}\cap B^{-}\\ \partial A^{0}\cap B^{1} & \partial A^{0}\cap \partial B^{1} & \partial A^{0}\cap B^{0} & \partial A^{0}\cap \partial B^{0} & \partial A^{0}\cap B^{-}\\ A^{-}\cap B^{1} & A^{-}\cap \partial B^{1} & A^{-}\cap B^{0} & A^{-}\cap \partial B^{0} & A^{-}\cap B^{-} \end{array}\right]$$

This $I_{5\times 5}M$ was presented in [18] and implemented in modeling cases of spatial relations between 3D spatial objects [31] and between fuzzy regions on a digital sphere [20]. However, to our knowledge, there is still no research work using $I_{5\times 5}M$ in modeling and computing topological relations among vague-shaped phenomena in distributed computing systems as in sensor networks. This research intends to adapt the $I_{5\times 5}M$ model to capture and explicitly describe topological relations that hold among vague-shaped phenomena monitored using a sensor network. Sensor nodes from their measurements can easily detect their locations as part of the kernel, conjecture and exterior of a given phenomenon. However, boundaries of the kernel and the conjecture parts cannot be inferred on a binary basis because sensor positions will eventually not match the exact edges of phenomena extent; each node detecting the proximity to a boundary may therefore hold an inner or outer position with respect to a boundary. 

**Figure 4:**
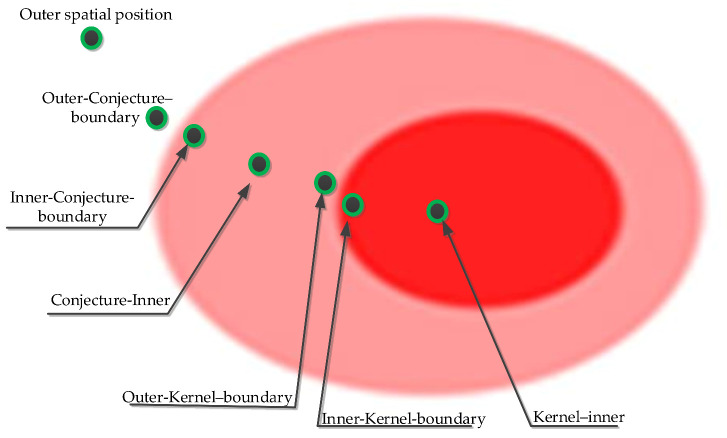
The seven possible relative spatial positions of sensor nodes towards the geometry of a monitored phenomenon.

For each monitored phenomenon, a sensor location will belong to the kernel or conjecture part if the phenomenon is detected, otherwise the sensor will be in the exterior of the phenomenon. The detection of phenomenon A (Kernel A or Conjecture A) by a sensor triggers the preparation and propagation of queries to one hop (directly linked) neighbors named N(s) through the communication mesh materialized by links between nodes. This collaboration among nodes aims at inferring spatial information about the extent and geometry (spatial boundaries) of phenomenon A. Then, to describe A’s geometry, seven spatial categories of sensor nodes can be identified, [24]. These categories are: kernel–inner; inner-kernel–boundary; outer-kernel–boundary; conjecture–inner; inner-conjecture–boundary; outer-conjecture–boundary; or outer, as shown Figure 4.

In Figure 4, the spatial state of sensor nodes is identified by the relative position of the sensor towards the different topological parts of the spatial object. 

### 3.1. The Conceptual Framework of the Proposed Approach

In multisensory networks, sensor nodes may use several sensing units to observe different phenomena in order to compute meaningful information [37]. The rapid evolution of microprocessors and nanofabrication techniques provides a basis for the rapid evolution of sensors [38]. Such sensors can compute meaningful information from sensed data, reduce the communication load to relevant information, and therefore reduce the consumption of energy [39]. 

**Figure 5:**
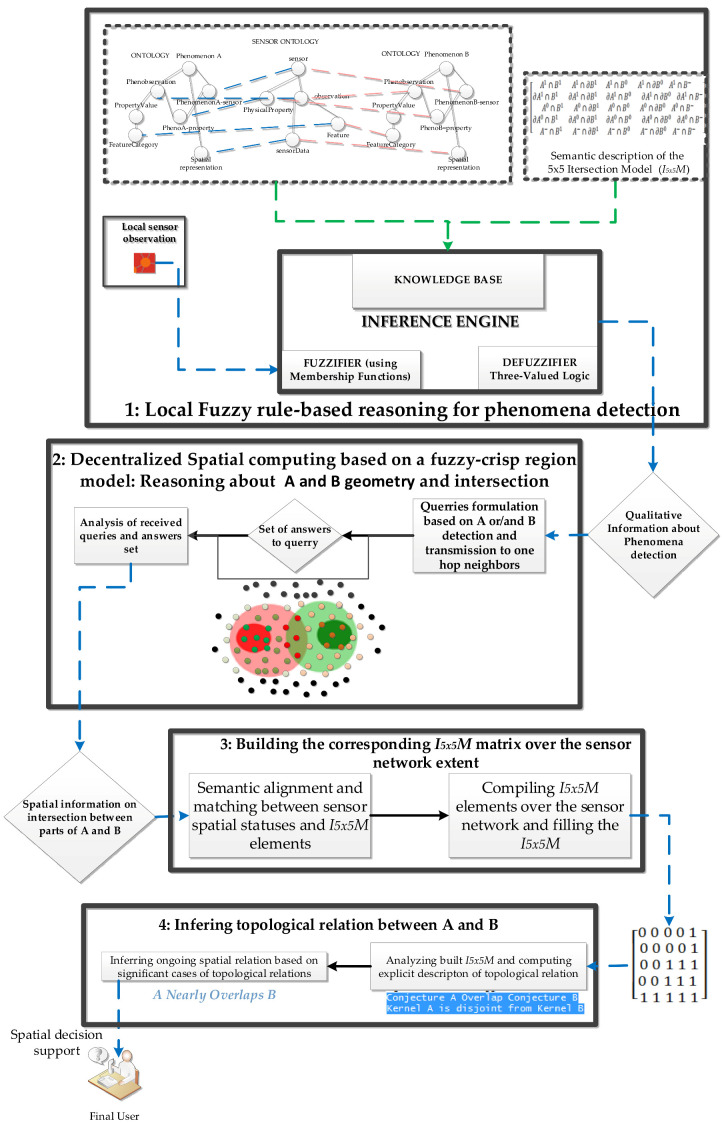
Conceptual framework for computing fuzzy spatial relations using $I_{5\times 5}M$ in SN.

The conceptual framework of our approach used in computing fuzzy spatial relations from sensor observation is made of four steps, as shown in Figure 5.

To compute fuzzy spatial relations that may hold between two sensed phenomena with vague boundaries, a sensing agent should be able to resolve the issue of fuzziness in observed properties while computing the detection of monitored phenomena [40] and spatial information about phenomena geometry and intersections through collaboration among linked nodes.

The proposed approach is composed of four main steps, which are:

1. Local fuzzy rule-based detection of monitored phenomena: in this step, sensor nodes use a built-in reasoning engine to evaluate the membership of their location to different parts of a monitored phenomenon (see Figure 4). This reasoning is based on the definition of a membership function (MF) and the semantics of observed data at a given time. Defuzzification rules based on three-valued logic are also set accordingly for each of the monitored phenomena (see Section 3.1.1).
2. Decentralized spatial computing based on a fuzzy-crisp region model: this stem is focused on fuzzy inference of spatial boundaries and local inference of intersection between monitored phenomena: each node collaborates with one-hop neighbors based on their phenomenon detections and the semantics of the adopted spatial model to infer their relative position to phenomenon boundaries (see Section 3.1.2).
3. Building the corresponding $I_{5\times 5}M$ matrix over the sensor network extent: this is completed by aggregating distributed spatial information about the intersection among sensed phenomena over the SN, by aligning local inferred intersection by sensors with $I_{5\times 5}M$ elements (see Section 3.1.3).

4. Inferring the topological relation that holds between two monitored phenomena: this is completed using the corresponding $I_{5\times 5}M$ explicit spatial description of a topological relation that holds among monitored phenomena, which is computed on a real-time basis (see Section 3.1.4).

#### 3.1.1. Fuzzy Rule-Based Detection of Monitored Phenomena

This is the first step of the proposed approach. Using a built-in reasoning engine, each sensor evaluates its membership to different parts of the spatial extent of monitored phenomena using membership functions (MFs) and the recorded data at a given time. MFs by definition cope with the semantics of sensor data and the ones of the monitored phenomena [41]. Considering the semantic description of a fuzzy-crisp object made of a kernel, conjecture and outside parts modeling monitored phenomena, the fuzzy set obtained from sensor data membership evaluation is transformed into a crisp set using three-valued logic to define IF-Then rules to be executed for defuzzification process [24]. From the defuzzification process, the fuzzy set made of sensor data is translated into a set of spatial qualitative values expressing if the sensor location belongs to the vague object representing the phenomenon (kernel or conjecture) or if it is out of the spatial extent of monitored phenomenon.

To compute local spatial information about the detection of sensed phenomena A and B, our approach uses two functions defined as follows:

- $f_{A}\left(s_{i}\right)\to \left\{\left(0,1\right)|\left(0,1,2\right)\right\}$
- $g_{B}\left(s_{i}\right)\to \left\{\left(0,1\right)|\left(0,1,2\right)\right\}$

functions $f_{A}\left(s_{i}\right)$ and $g_{B}\left(s_{i}\right)$ are defined for the detection of phenomenon A and B, respectively, by a given sensor $\left(s_{i}\right)$ in accordance with their semantics. Their images are represented on two bits where the first bit expresses the detection (value = 1) or non-detection (value = 0); the second bit defines to which topological part of the spatial extent of a detected phenomenon the location of $\left(s_{i}\right)$ belongs; this is defined as follows: 0 → external (only when not detected); 1 → kernel and 2 → conjecture.

The second step in our approach is devoted to decentralized reasoning for boundaries detection and intersection analysis between monitored phenomena by sensors.

#### 3.1.2. Decentralized Spatial Computing Based on a Fuzzy-Crisp Region Model 

This is the second step of the approach. A node detecting the monitored phenomenon can guess border detection but will not identify boundary vertices with certainty. A third function is used to define boundary detection for each of a monitored phenomenon by a node $\left(s_{i}\right)$.

- $h\left(s_{i}\right)\to \left\{0,1,2\right\}$

Its image is represented on a third bit using three values as follows: 0 → no boundary detected, 1 → inner boundary position and 2 → outer boundary position. These relative boundary positions are set for the kernel and the conjecture part. In this way, seven different sensor states describing the fuzzy spatial extent of a phenomenon are coded using three bits as follows: [0 0 0] for outer nodes, [1 1 0] for kernel inner nodes, [1 1 1] for kernel inner boundary nodes, [1 1 2] for kernel outer boundary nodes, [1 2 0] for conjecture inner nodes, [1 2 1] for conjecture inner boundary nodes and [1 2 2] for conjecture outer boundary nodes (see Figure 4).

For the perspective of building the $I_{5\times 5}M$ modeling the fuzzy spatial relation that holds between A and B, boundary indeterminacy is solved by using the lower or upper fuzzy approximation suggested in [42]. In our approach, we choose the lower approximation and consider the inner boundary position of kernel or conjecture part as the boundary positions. This approximation allows us to omit the inner and outer boundary spatial states in favor of a single boundary spatial state. The 25 relevant states which may be obtained by sensors from the inferred geometry of two monitored phenomena and coded on six bits are presented in Table 1.

The $I_{5\times 5}M$ modeling evolving fuzzy spatial relations are populated by relevant sensors spatial states from these 25 states when inferred from sensor measurements and collaborations. 

#### 3.1.3. Building the Corresponding $I_{5\times 5}M$ Matrix over the Sensor Network Extent

Aggregating the whole set of partial spatial information computed over the SN extent is required for building a holistic view about the observed phenomena and inferring explicit knowledge about the fuzzy spatial relation that holds among them. From this third step of the approach, the I_5×5_ M modeling the fuzzy spatial relation that holds in the monitored space is built by aggregating distributed spatial information on the intersection of A and B, computed over the SN. Information exchange and aggregation in SN follow the network topology and should respect a routing protocol to avoid redundant and useless transmission of information for energy economy and fast delivery [43]. 

An appropriate route is required for the optimum transmission of information from/to each node to/by the sink to ensure minimum transmission of information by intermediate nodes and therefore an economy of energy and fast delivery [43]. The minimum spanning tree (MST), which determines for every node the optimum route to the target (sink/sensor node), should be established from the communication network which is theoretically represented following the RNG (Relative Neighborhood Graph) or GG (Gabriel Graph) graphs [43]. These two graphs are built based on sensor spatial range (communication or observation) whose extent corresponds to the diameter of the containing circle; the RGN graph does not allow any overlapping among the containing circle, while the GG graph does. The MST is used during the propagation of information, instructions or queries among sink and sensor nodes. Each node therefore knows its children nodes and also its parent node through which the final spatial information to be transmitted to the sink should be conveyed.

A sink node is elected among the sensors of the network; the sink node stores an $I_{5\times 5}M$, whose elements are all set to 0 corresponding to an empty set (Ø). From the detection of monitored phenomena and their geometries, each node set its spatial state, which is in alignment with an item of the $I_{5\times 5}M$ identified as $I_{5\times 5}M_{i,j}$, where i and j correspond to the number of the line and column. If a sensor’s spatial state is equal to: [1 1 1][1 1 1], the sensor will send a message containing $I_{5\times 5}M_{1,1}=1$ to its parent over the spanning tree. This message corresponds to $A^{1}\cap B^{1}\neq \emptyset ==I_{5\times 5}M_{1,1}=1$ as illustrated in Equation (4) for a given sensor Si.

(4) $$\mathrm{If}\;\mathrm{spatial}\_\mathrm{state}\;(\mathrm{Si})=[1\;1\;1][1\;1\;1]\;\mathrm{then}\;A^{1}\cap B^{1}\neq \emptyset ==I_{5\times 5}M_{1,1}=1$$

Over the spanning tree branches linking nodes to the sink node, the sensor spatial states, translated into $I_{5\times 5}M_{i,j}$ elementary values, are aggregated from the end child node to the last parent node (sink). Redundant values are ignored during the aggregation process; the set of $I_{5\times 5}M_{i,j}\;$ elementary values collected over the spanning tree at a given time is inserted in $I_{5\times 5}M$ while the others remain with a value of zero. Such filled $I_{5\times 5}M$ represents the fuzzy spatial relation that holds between phenomena A and B and their parts. Hence, the structure of the matrix can be used for inferring an explicit spatial description of the fuzzy spatial relation that holds between monitored phenomena.

#### 3.1.4. Inferring the Topological Relation That Holds between Two Monitored Phenomena 

Topological relations describe how humans perceive spatial configurations between objects in reality and they constitute a significant part of information in human communication by natural language. We may say, for instance, that a certain geographic region is adjacent to, contained in or overlapping with another [44]. If we consider two vague-shaped regions A and B involved in a fuzzy spatial relation, we may use the taxonomy of fuzzy spatial relations proposed by Clementini and Di Felice [33]. This taxonomy is a good communication tool but it lacks specific information that can help the end user in building an appropriate image of the monitored reality. For instance, saying A meets or nearly overlaps B may not provide enough information to the end user to have a good picture of the scene as is illustrated in Figure 2. Taking advantage of the fine observation of sensors and their ability to infer meaningful spatial information, we may further describe this using other topological information as:

- Conjecture of A overlaps the conjecture of B
- Conjecture of A overlaps the kernel and conjecture of B
- Conjecture of B overlaps the kernel and conjecture of A
- Conjecture of A overlaps the kernel and conjecture of B, and the kernel of A overlaps conjecture of B
- Kernel of A is disjoined from the kernel of B, or the conjecture of A is disjoined from conjecture of B

**Figure 6:**
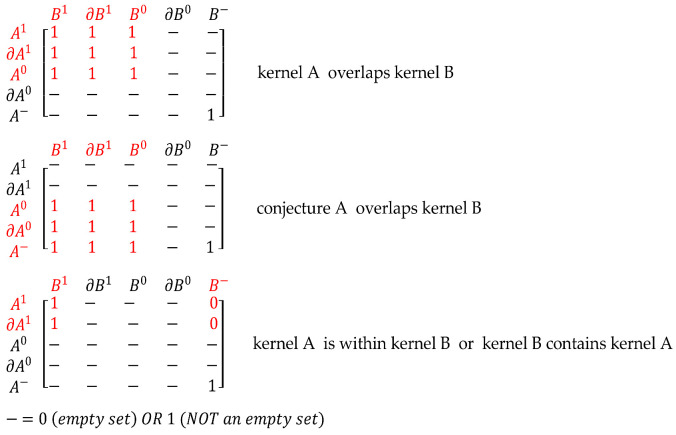
Illustration of three cases of fuzzy spatial relations between topological parts of A and B sensed phenomena, inferred from $I_{5\times 5}M$ structure.

Using $I_{5\times 5}M$, one can say region A meets region B, providing users with qualitative information to describe the evolving spatial relations between A and B, which is relevant for SDSS and many other GIS applications. With the proposed approach, we will be able to derive such qualitative information describing inferred fuzzy spatial relations using $I_{5\times 5}M\;$ elements. For this purpose, some reasoning rules are set on the basis of $I_{5\times 5}M\;$ structure to infer if a topological part of A overlaps or contains another topological part of B and vice versa. In the following examples, three cases of fuzzy spatial relations between topological parts of A and B extracted using the components of $I_{5\times 5}M$ are presented. The reasoning rules for the specific relations illustrated in Figure 6 are presented as follows:

If $M=I_{5\times 5}M$, the reasoning rules can be formulated as follows for illustration:

- If $M\left(1,1\right)+M\left(2,2\right)+M\left(3,3\right)=3$, then kernel A overlaps kernel B (this means, for example, that the intersections kernel of A with kernel of B; boundary of kernel of A with boundary of kernel of B and exterior of kernel of A with exterior of kernel of B are not empty sets ie ≠ Ø)

- If $M\left(5,1\right)+M\left(4,2\right)+M\left(3,3\right)=3$, then conjecture A overlaps kernel B

- If $M\left(5,5\right)+M\left(4,4\right)+M\left(3,3\right)=3$, then conjecture A overlaps conjecture B

- If $M\left(1,5\right)+M\left(2,4\right)+M\left(3,3\right)=3$, then kernel A overlaps conjecture B

It should be noted that overlapping excludes the containing or covering relation between two parts; that being said, if the predicate overlap is set as true between kernel A and kernel B, the predicate contain or cover cannot be set as true between these two parts.

Evaluating the relation of the type contain or cover between parts of A and B depends on $I_{5\times 5}M$ units expressing the intersection of any part of A or B and the exterior of the related phenomenon. For instance: if $A^{1}\cap B^{-}=\;Ø$ and $\partial A^{1}\cap B^{-}=\;Ø$, the kernel of A is contained by a given part of B. Identifying which part of B contains the kernel A is necessary; if kernel A overlaps kernel B then we can check if the conjecture of B contains kernel A: $A^{1}\cap B^{0}\neq \;Ø$ and $\partial A^{1}\cap B^{0}\neq \;Ø$.

These rules can be formally written as follows:

- **If** $\left(M\left(1,5\right)+M\left(2,5\right)=0\right)\;and\;\left(M\left(1,1\right)+M\left(2,2\right)+M\left(3,3\right)\neq 3\;\right)\;and\;$$\left(M\left(1,1\right)+M\left(2,1\right)=2\right)\;then\;kernel\;B\;covers\;kernel\;A$
- **If** $\left(M\left(1,5\right)+M\left(2,5\right)=0\right)\;and\;\left(M\left(1,1\right)+M\left(2,2\right)+M\left(3,3\right)\neq 3\;\right)\;and\;$$\left(M\left(1,1\right)+M\left(2,1\right)\neq 2\right)\;and\;\left(M\left(1,3\right)+M\left(2,3\right)\;=\;2\right)\;then\;conjecture\;B\;covers\;kernel\;A$

Kernel A and kernel B will be called disjoint if the overlap predicate is set as false and predicate cover or contain is set as false also. The rule used to evaluate the disjunction between kernel A and kernel B is formalized as follows:

- **If**$\left(M\left(1,5\right)+M\left(2,5\right)=2\right)\;and\;\left(M\left(5,1\right)+M\left(5,2\right)=2\right)\;and\;\left(M\left(1,1\right)+M\left(2,2\right)+M\left(3,3\right)\neq 3\right)\;$then kernel A is disjoint kernel B

Equality between parts is established if the overlap predicate is **true** and the **cover** or **contain** predicate is **jointly true** for the two parts, but the containing or covering parts are jointly undefined; equality jointly involves overlapping and parthood as shown in the conceptual neighborhood graph of RCC8 [45]. For equality between kernel A and kernel B, the reasoning rule can be set as follows:

- **If**$\left(M\left(1,5\right)+M\left(2,5\right)=0\right)\;and\;\left(M\left(5,1\right)+M\left(5,2\right)=0\right)\;and\;\left(M\left(1,1\right)+M\left(2,2\right)+M\left(3,3\right)=3\right)\;and\;\left(M\left(1,1\right)+M\left(1,2\right)\neq 2\right)\;and\;\left(M\left(1,1\right)+M\left(2,1\right)\neq \;2\right)\;then\;kernel\;A\;is\;equal\;to\;kernel\;B$

Such rules are formulated for other parts of fuzzy-crisp regions A and B based on the $I_{5\times 5}M$ structure presented in Equation (3). The formal presentation of the approach is broadly presented in the next section.

The detailed structure of $I_{5\times 5}M$ is used for providing a more explicit description of the inferred topological relation from sensor network data for more effective support to SDSS. This constitutes a major contribution of this research.

### 3.2. Formal Presentation of Proposed Approach

As presented earlier, sensors need to collaborate with their linked neighbors and use a built-in reasoning engine to infer partial spatial information on the monitored phenomena of vague spatial shape. This reasoning process allows us to start work on fine grain spatial information observed by the individual sensors nodes in Sn and infer topical relations between the observed phenomena through an aggregation process using the elements of information in the $I_{5\times 5}M$. Hence, the sink node in SN uses a built-in reasoning engine to compute spatial relation modeled using the information in $I_{5\times 5}M$. The whole process described in former sections is summarized in the following algorithm presented in Algorithm 1.
**Algorithm 1** Fuzzy spatial relation modeling by *I*_5×5_*M* in sensor networks**1:****Variables*: SN****= **{s1,s2,...,sn}**; SensorNetwork*2:
    *S-On, D-Ons; a sensor ontology, a domain ontologies*
3:    *I*_5×5_
*M; Intersection matrix modeling the fuzzy spatial relation between A and B*
4:    ***f_A_(s_i_)** →*
**{(0,1)|(0,1,2)}**
*; Function used to detect A*
5:      ***g_B_(s_i_)** →*
**{(0,1)|(0,1,2)}**
*; Function used to detect B*
6:      ***h(s)→***
**{0,1,2}**
*; Function used to detect the boundaries of A or B*
7:      ***SpTr(SN);** Built spanning tree over the sensor network*
8:**Begin**9:      *Setting the sensor network; establishing communication links among nodes*
10:      ***Build the spanning tree (SpTr(SN))** over the SN topology*
11:      *Detection of the phenomenon A is computed over the SN: **f_A_(SN)***
12:      *Detection of phenomenon B is computed over theSN: **g_B_(SN)***
13:***Decentralized spatial computing****of A and B **boundaries** over the **SN:h(s_i_(AB))***14:***Compute spatial state****from detected phenomena and boundaries: **(s_i_*****([*x,y,z*][*x y z*])**15:***Aggregate****sensor spatial state*16:      ***From the** last child node to sink node along **SpTr(SN)***
17:      ***Compile** spatial states ofunequal value*
18:      ***Compute I*****_5×5_*M***
*by alignment between spatial states values and **I*****_5×5_*M***
*elements*
19:***If...then****Inference ofqualitative specifications about fuzzy spatial relationfrom **I*****_5×5_*M***20:**End.**

Evaluating the performance of this approach and its applicability is a priority of this research. For this purpose, the corresponding computer program was implemented within a multi-agent programming software named Netlogo.

## 4. Case Study and Results

The program, developed here with Netlogo, loads geospatial scenes made of vague-shape phenomena; sensors equipped with a built-in reasoning engine compute the detection of phenomena and their boundaries. From these boundaries, the nature of relations that holds between detected phenomena is computed. These relations are modeled with $I_{5\times 5}M$, from which qualitative spatial specifications about these relations are inferred. The 44 realizable fuzzy relations between vague-shape spatial objects [46] are adapted for reasoning with sensor data and are implemented in this research.

As a first case study, we use sensor data on two vague-shaped phenomena observed by an SN. These phenomena include a bush fire (A in red color for the kernel and light red for the conjecture) and wild shrubby vegetation (B with green color and light green for the kernel and conjecture, respectively). The SN has a sink node and sensor nodes that receive information from other sensors in the network through their communication systems. 

**Figure 7:**
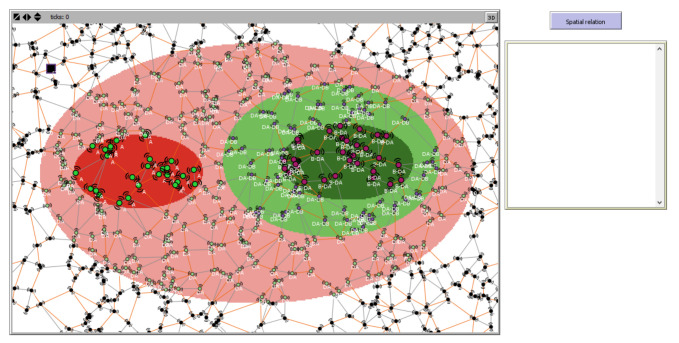
Excerpt of Netlogo environment showing A’s boundary covering B within SN extent.

Without boundary detection, it is not possible to infer spatial information about the relation between A and B. This is why the output of the spatial relation reasoning section on the right of the figure is empty, as shown in Figure 7; after boundaries detection, computing the spatial relation between A and B will yield the corresponding $I_{5\times 5}M$ and qualitative spatial specification about the relation between A and B.

In Figure 7, black lack nodes represent sensors in the outer part of the phenomena while colored nodes express the detection of the phenomena. In addition, the differences in sizes of the nodes express the parts of spatial extent of the observed phenomena (kernel, conjecture). The lines represent communication links, and when colored in orange, the links are part of the spanning tree.

**Figure 8:**
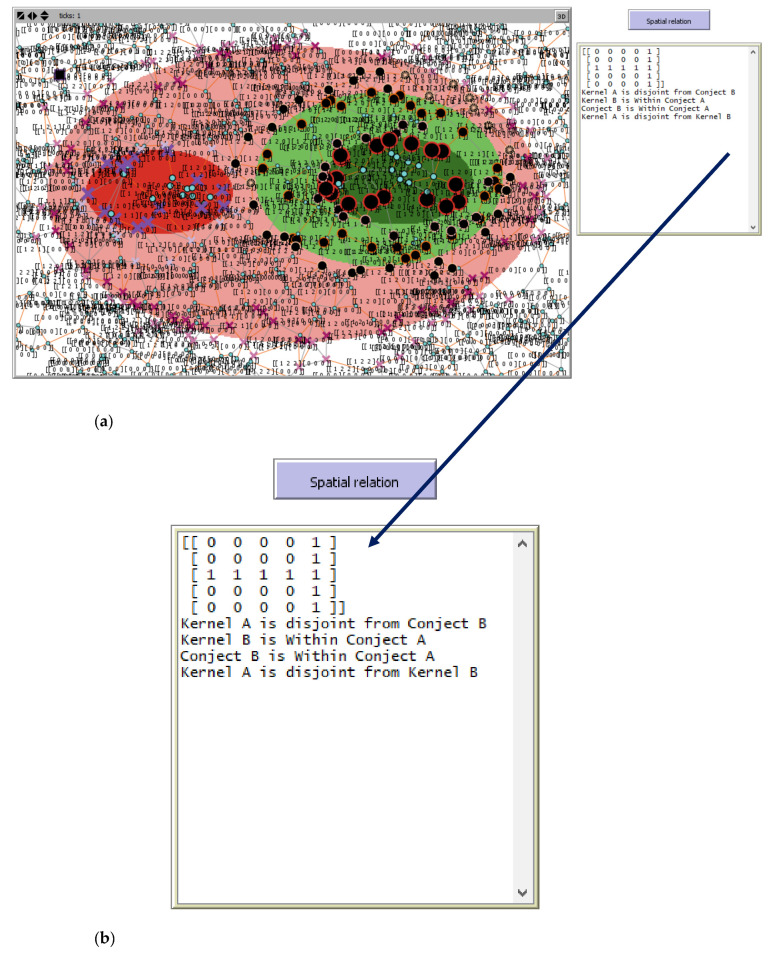
(**a**) The SN showing sensor spatial states as the sensor label after boundary detection and intersection analysis and (**b**) enlargement of the output showing resulting $I_{5\times 5}M$ and explicit describing the fuzzy spatial relation.

The output of decentralized spatial reasoning for boundaries detection changes the shape of nodes detecting phenomena boundaries, and the $I_{5\times 5}M$ elements are aligned with the sensors’ spatial state; updated $I_{5\times 5}M\;$ is inserted in the output area followed by a spatial description of the scene computed from $I_{5\times 5}M$ structure and elements value; this is shown in Figure 8.

The approach yields compelling results for all the 44 realizable fuzzy spatial relations; this was effective for an SN in which sensors were adequately distributed over the spatial extent of the observed spatial scene to enable the detection of any spatial dynamic phenomena and their interactions over the scene. The results obtained for the 44 realizable fuzzy relations identified in [46] are presented in Appendix A. This Appendix A gives, for each type of fuzzy spatial relation, a view of the corresponding scene from which the derived $I_{5\times 5}M$ is given with the inferred description of the monitored scene.

## 5. Discussion

Former research on decentralized computation on the spatial relationships between sensed phenomena was based on RCC8 (eight spatial relations) and did not consider the possible vague shape and broad boundaries of sensed phenomena [47]. The proposed approach took advantage of the $I_{5\times 5}M$ structure to consider the fuzzy nature of real-world phenomena and could consistently model 44 cases of topological relations that can hold between sensed phenomena of vague shapes. Inferred descriptions of evolving spatial relationships from built $I_{5\times 5}M$ give an opportunity to redraw a spatiotemporal representation of monitored reality that can enhance spatial decision support systems. 

From detected phenomena, our approach can build the corresponding $I_{5\times 5}M$ from which an explicit description of the monitored scene with the presence of vague spatial phenomena and their interactions is inferred on a near real-time basis. Instead of just declaring the nature of the relation between sensed phenomena, the provided description may help in depicting the scene for decision-making processes. For instance, instead of just saying A and B are strongly overlapping because their kernels overlap each other, their conjectures may either be equal, overlapping, or one is contained in the other. Such an inferred description is necessary in guiding robots or autonomous engines over sensed phenomena.

**Figure 9:**
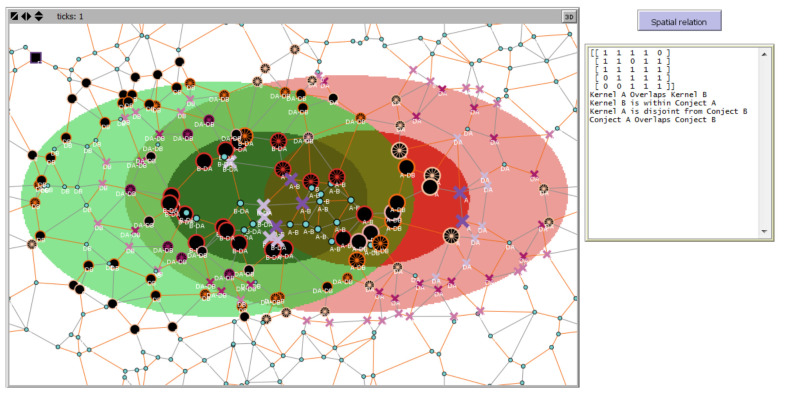
Poor modeling and qualitative specifications about a given scene monitored by a light dense SN of wide range sensors (case of scene 19).

The use of a wide-range sensor lightly distributed over the area of interest where observations are carried may result in some imprecision in inferred spatiotemporal information from which reasoning about the spatial relation is carried, therefore yielding poor and imperfect spatial knowledge, as shown in Figure 9. 

Such a poorly distributed sensor network may not capture the essential spatial information about the intersection between monitored phenomena and yield a $I_{5\times 5}M$ which does not conform to a real-world scene. This may lead to poor and inadequate spatial decisions. For many applications, it is required that each point of the region is covered by at least k (k > 1) sensors [48]; in our case, the spatial distribution of sensors should be set in order to assure that all relevant information depicting an ongoing scene is captured. Adequate spatial distribution of sensors on the field, concomitantly depends on the performance of sensors (observation range), their relative position and their density. A GIS-based approach for optimal distribution and positioning of sensors was developed by Argany and Mostafavi in [49], which can be combined with the proposed approach in this paper to improve the coverage of the SN as well as its efficiency in monitoring and detecting the information and events of interest in a given spatial scene. 

On the other hand, some real-world phenomena may be adequately represented using more complex fuzzy-crisp object models. For instance, we may consider cases that objects may have several kernel parts or include islands. Modeling and analyzing topological relations that hold between such phenomena using $I_{5\times 5}M$ was not tested in this research. Future investigations will also concern reasoning and computing continuous change in topological relations between monitored phenomena in sensor networks.

Finally, it should be noted that the implementation and integration of the proposed algorithm in a specific type of sensor were not explored in this study. Further investigations would be necessary to estimate to what extent we need to optimize the operations so that each sensor node is able to carry out all the proposed operations for continuously inferring qualitative information on spatial relations of detected phenomena without the burden on its energy consumption and communication capacities. This project is a part of ongoing research on sensor network deployment and exploitation in natural and built environments and further progress on our results will be reported in upcoming publications.

## 6. Conclusions and Future Works

This research has given us the opportunity to develop a decentralized fuzzy rule-based approach for sensor networks for reasoning about fuzzy spatial relations and interactions between two monitored spatial dynamic phenomena uncertain boundaries. In this approach, we assumed that multi-sensors and a sink node were organized in an SN and are equipped with a built-in reasoning engine enabling effective detection of at least two monitored phenomena modeled with a fuzzy-crisp spatial model whose geometries are made of kernel and conjecture parts. The fuzzy-crisp object adopted in this research, as a best representation of vague phenomena, is composed of five topological parts including the kernel, the conjecture and the exterior, and their boundary zones from which the 5 × 5 intersection model ($I_{5\times 5}M$) is built. The use of an $I_{5\times 5}M$ helps in modeling the possible spatial relation based on the intersection between the five topological parts of two vague-shaped phenomena that are observed by an SN. Our proposed original approach allows inferring qualitative specifications about the captured spatial relation between two vague-shaped phenomena at a given time from the structure of the resulting $I_{5\times 5}M$. This provides valuable information for an SDSS which can enable any user or intelligent agent to easily analyze the real-time situation and decide on the actions to be taken. Testing the proposed approach yielded compelling results for the 44 realizable fuzzy spatial relations that may hold between two given phenomena with a vague shape monitored by SN.

In future work, it will be interesting to consider the implementation of the proposed algorithm in SN composed of sensors with nano-computing capacities and test it in a real-world application. The evaluation of the performance of such a network with an optimal placement of its sensor nodes would be another interesting avenue for further investigations.

## Figures and Tables

**Table 1:** Possible sensor spatial states.

Sensor Spatial State	Spatial Meaning	Sensor Spatial State	Spatial Meaning
[0 0 0][0 0 0]	Exterior (A) ∩ Exterior (B) ≠ Ø	[1 1 1][1 1 0]	Kernel boundary (A) ∩ kernel (B) ≠ Ø
[1 1 0][0 0 0]	kernel (A) ∩ Exterior (B) ≠ Ø	[1 1 1][1 1 1]	Kernel boundary (A) ∩ Kernel boundary (B) ≠ Ø
[1 1 1][0 0 0]	Kernel boundary (A) ∩ Exterior (B) ≠ Ø	[1 1 1][1 2 0]	Kernel boundary (A) ∩ conjecture (B) ≠ Ø
[1 2 0][0 0 0]	conjecture (A) ∩ Exterior (B) ≠ Ø	[1 1 1][1 2 1]	Kernel boundary (A) ∩ conjecture boundary (B) ≠ Ø
[1 2 1][0 0 0]	Conjecture boundary (A) ∩ Exterior (B) ≠ Ø	[1 2 0][1 1 0]	Conjecture (A) ∩ kernel (B) ≠ Ø
[0 0 0][1 1 0]	Exterior (A) ∩ kernel (B) ≠ Ø	[1 2 0][1 1 1]	Conjecture (A) ∩ kernel boundary (B) ≠ Ø
[0 0 0][1 1 1]	Exterior (A) ∩ kernel boundary(B) ≠ Ø	[1 2 0][1 2 0]	Conjecture (A) ∩ conjecture (B) ≠ Ø
[0 0 0][1 2 0]	Exterior (A) ∩ conjecture (B) ≠ Ø	[1 2 0][1 2 1]	Conjecture (A) ∩ conjecture boundary (B) ≠ Ø
[0 0 0][1 2 1]	Exterior (A) ∩ conjecture boundary (B) ≠ Ø	[1 2 1][1 1 0]	Conjecture boundary (A) ∩ kernel (B) ≠ Ø
[1 1 0][1 1 0]	kernel (A) ∩ kernel (B) ≠ Ø	[1 2 1][1 1 1]	Conjecture boundary (A) ∩ kernel boundary (B) ≠ Ø
[1 1 0][1 1 1]	kernel (A) ∩ kernel boundary (B) ≠ Ø	[1 2 1][1 2 0]	Conjecture boundary (A) ∩ Conjecture (B) ≠ Ø
[1 1 0][1 2 0]	kernel (A) ∩ conjecture (B) ≠ Ø	[1 2 1][1 2 1]	Conjecture boundary (A) ∩ Conjecture boundary (B) ≠ Ø
[1 1 0][1 2 1]	kernel (A) ∩ conjecture boundary (B) ≠ Ø		

## Data Availability

Not applicable.

## Appendix A

**Table A1:** Spatial Modeling of 44 Fuzzy Spatial Relations Using the $I_{5\times 5}M$.

Scene					
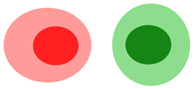
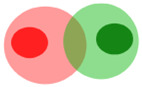
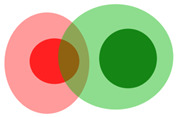
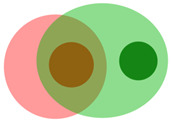
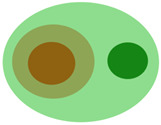
Resulting $I_{5\times 5}M$ and inferred spatial description of the relation					
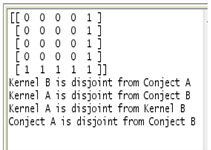
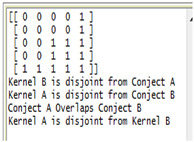
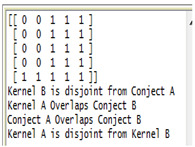
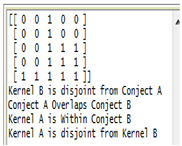
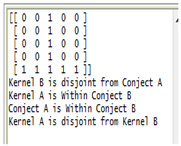
Corresponding spatial relation	1: Disjoint	2: Meet	3: nearly Overlap	4: covered by Boundary	5: covered by Boundary
Scene					
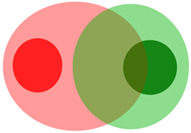
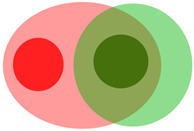
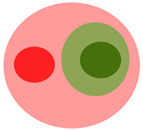
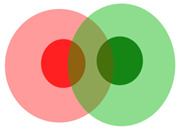
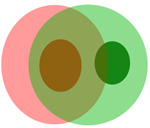
Resulting $I_{5\times 5}M$ and inferred spatial description of the relation					
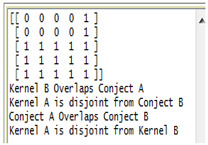
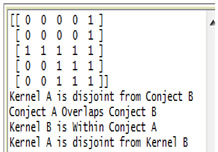
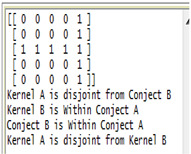
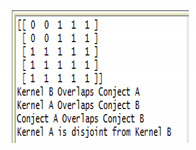
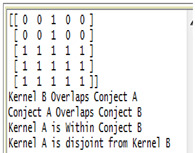
Corresponding spatial relation	6: nearly Overlap	7: covers with Boundary	8: covers with Boundary	9: nearly Overlap	10: covered by Boundary
Scene					
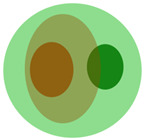
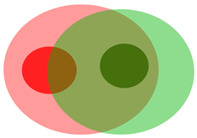
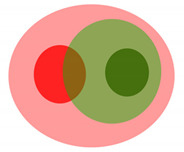
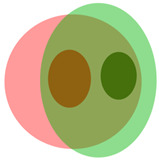
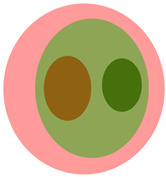
Resulting $I_{5\times 5}M$ and inferred spatial description of the relation					
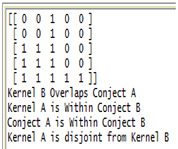
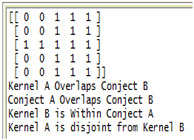
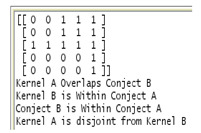
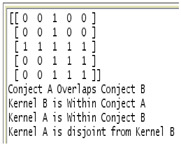
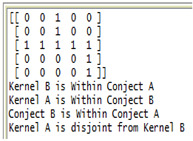
Corresponding spatial relation	11: covered by Boundary	12: covers with Boundary	13: covers with Boundary	14: boundary Overlap	15: boundary Overlap
Scene					
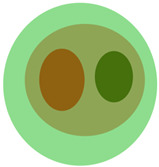
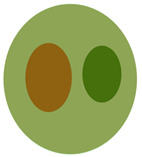
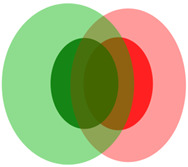
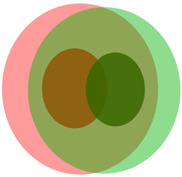
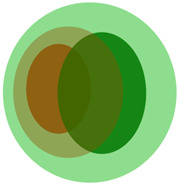
Resulting $I_{5\times 5}M$ and inferred spatial description of the relation					
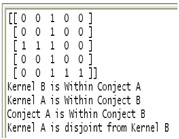
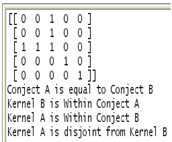
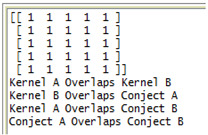
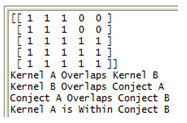
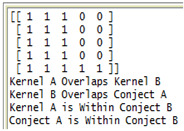
Corresponding spatial relation	16: boundary Overlap	17: boundary Overlap	18: Overlap	19: nearly Covered by	20: nearly Covered by
Scene					
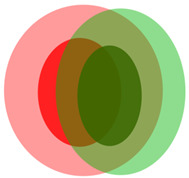
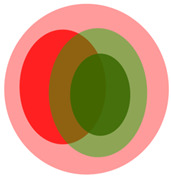
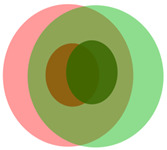
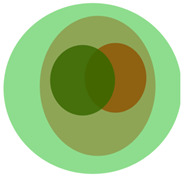
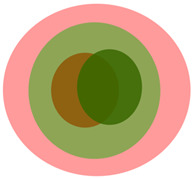
Resulting $I_{5\times 5}M$ and inferred spatial description of the relation					
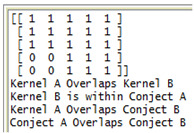
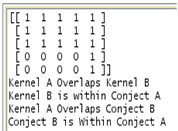
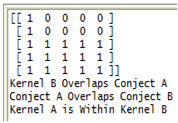
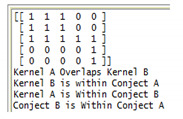
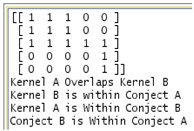
Corresponding spatial relation	21: nearly Covers	22: nearly Covers	23: strongly Overlap	24: strongly Overlap	25: strongly Overlap
Scene					
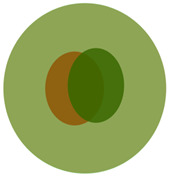
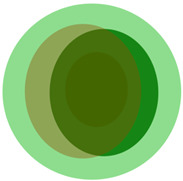
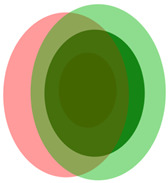
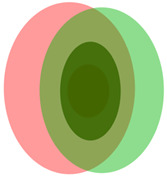
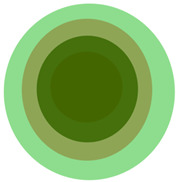
Resulting $I_{5\times 5}M$ and inferred spatial description of the relation					
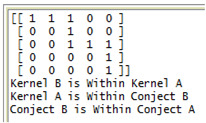
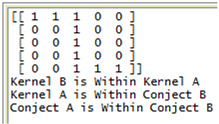
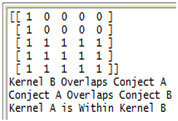
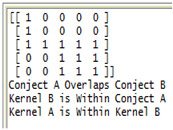
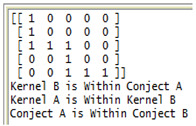
Corresponding spatial relation	26: strongly Overlap	27: covered by	28: nearly Covered by	29: nearly Fill	30: nearly Fill
Scene					
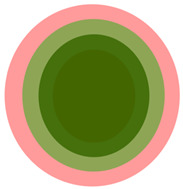
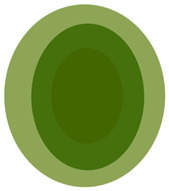
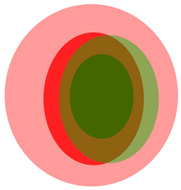
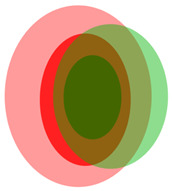
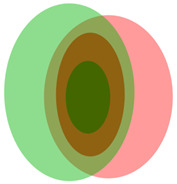
Resulting $I_{5\times 5}M$ and inferred spatial description of the relation					
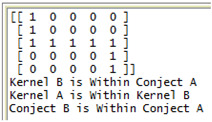
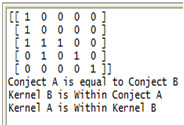
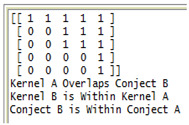
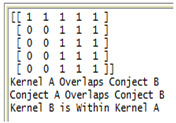
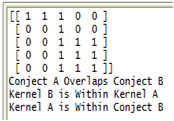
Corresponding spatial relation	31: nearly Fill	32: nearly Fill	33: covers	34: nearly Covers	35: nearly Filled by
Scene					
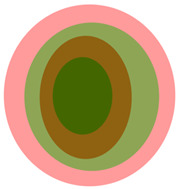
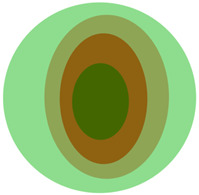
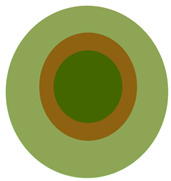
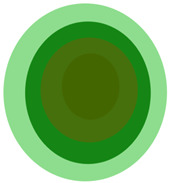
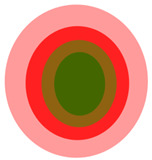
Resulting $I_{5\times 5}M$ and inferred spatial description of the relation					
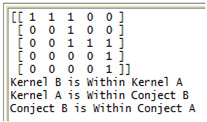
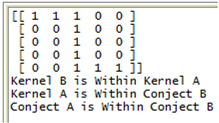
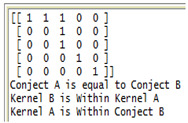
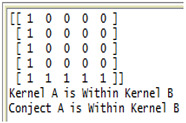
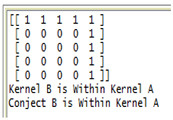
Corresponding spatial relation	36: nearly Filled by	37: nearly Filled by	38: nearly Filled by	39: Inside	40: Contains
Scene					
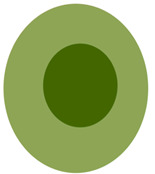
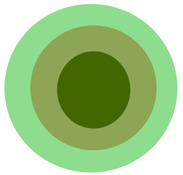
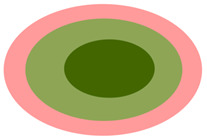
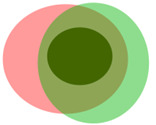
Resulting $I_{5\times 5}M$ and inferred spatial description of the relation					
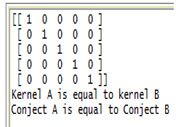
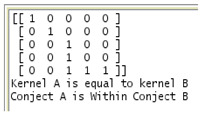
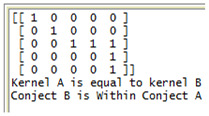
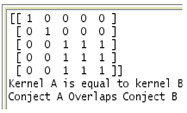
Corresponding spatial relation	41: Equal	42: nearly Equal	43: nearly Equal	44: nearly Equal	
